# Gambogic acid induces cell death via covalent binding with PRDX1 to regulate ER stress and autophagy

**DOI:** 10.1186/s13020-025-01144-8

**Published:** 2026-06-03

**Authors:** Jinyan Wang, Wei Zhang, Li Yang, Xiaoru Zhong, Jinhuan Ou, Ashok Iyaswamy, Xin Gu, Chuanbin Yang, Bing Guo, Mingjun Shi, Jigang Wang

**Affiliations:** 1 Department of Pathophysiology, Guizhou Medical University, Guiyang, Guizhou China; 2 Center for Drug Research and Development, Guangdong Provincial Key Laboratory for Research and Evaluation of Pharmaceutical Preparations, Guangdong Pharmaceutical University, Guangzhou, Guangdong China; 3 Department of Critical Care Medicine, Guangdong Provincial Clinical Research Center for Geriatrics, Shenzhen Clinical Research Center for Geriatrics, Shenzhen People’s Hospital (The First Affiliated Hospital, Southern University of Science and Technology, The Second Clinical Medical College, Jinan University), Shenzhen, Guangdong China; 4 Mr. & Mrs. Ko Chi-Ming Centre for Parkinson’s Disease Research, School of Chinese Medicine, Hong Kong Baptist University, Hong Kong SAR, China; 5 Department of Biochemistry, Karpagam Academy of Higher Education, Coimbatore, India; 6 State Key Laboratory for Quality Ensurance and Sustainable Use of Dao-di Herbs, Artemisinin Research Center, and Institute of Chinese Materia Medica, China Academy of Chinese Medical Sciences, Beijing, 100700 China

**Keywords:** Chemical proteomics, Gambogic acid (GA), Autophagy, Drug targets, PRDX1

## Abstract

**Background:**

Gambogic acid (GA) is a natural bioactive compound derived from Garcinia *hanburyi Hook. F*, has proven anticancer activity and is currently in Phase II clinical trials for the treatment of cancer. However, the molecular mechanisms and targets underlying GA's anti-renal cell carcinoma effects remain unclear.

**Methods:**

CCK-8 assay evaluated kidney cancer cell viability via formazan formation. Flow cytometry identified apoptotic cells using characteristic fluorescence signals. Western blotting assessed autophagy- and apoptosis-related protein expression through extraction, SDS-PAGE, transfer, and antibody detection. JC-1 assay determined mitochondrial health by measuring red-to-green fluorescence ratio in kidney cancer cells. The production of reactive oxygen species (ROS) was quantified through CM-H2DCFDA staining. Additionally, a range of techniques, such as proteomics, activity-guided protein profiling (ABPP), and cellular thermal stability assays (CETSA), were employed to ascertain the molecular targets involved.

**Results:**

GA induces cell death by inducing ER stress and modulating autophagy. GA-induced autophagy is involved in ER stress activation. Importantly, GA-induced ER stress and subsequently cell death is caused by increased ROS levels. Mechanistic studies show that peroxiredoxin-1 (PRDX1), a key antioxidant enzyme, is a directly covalent binding target of GA, and overexpression of PRDX1 mitigates GA-induced ROS production and subsequently cell death.

**Conclusion:**

The study identified PRDX1 is a potential directly covalent binding protein of gambogic acid, and elucidated its mechanism for inducing cell death that involving in ROS-mediated ER stress and autophagy regulation. The results obtained offer fresh perspectives on the mechanisms underlying the cytotoxic effects induced by gambogic acid, further suggesting that PRDX1 may represent a promising candidate for targeted therapy in the development of novel anticancer agents, notably for the treatment of kidney cancer.

## Introduction

Natural products isolated from herbal medicine have been a valuable resource for anti-cancer drug discoveries. GA (C_38_H_44_O_8_, MW 628.76 g/mol), a polyisopentenyl xanthone from Garcinia cambogia, shows nM-range anti-tumor activity against various cancers, highlighting its potential in cancer treatment and research [1]. GA exerts its antitumor effects through multiple molecular mechanisms, including inducing apoptosis [2], inhibiting the cell cycle [3], blocking angiogenesis [4], triggering ferroptosis [5], and modulating immune responses [6]. Breakthrough in the anti-cancer studies mainly focused on breast, lung, and liver cancers, while knowledge about the effects of GA in renal cancer cells remains largely unclear [7–9]. Renal cell carcinoma (RCC) ranks among the most prevalent urological malignancies, accounting for 2–3% of global cancer incidence while demonstrating an aggressive clinical course, with mortality rates reaching 30–40% [10]. Currently, the treatment strategies for RCC involve a multidisciplinary and multimodal comprehensive approach, including surgery, targeted therapy, immunotherapy, chemotherapy, and personalized combination therapy [11]. Both in vitro and in vivo research has shown that GA effectively halts tumor growth by targeting various molecules, such as proteins belonging to the B-cell lymphoma 2 (Bcl-2) family [12], thioredoxin-1/2 (TRX-1/2) and thioredoxin reductase 1 (TrxR1) [13, 14], inhibitory kappa B kinase (IKK) [15], heat shock protein 90 (Hsp90) [16], steroid receptor coactivator-3 (SRC-3) [17], and the transferrin receptor (TfR) located on the cell surface [18]. The collective effects of these targeted actions contribute significantly to the suppression of numerous tumor types. Despite notable advancements in pinpointing the GA's anticancer properties, its potential direct binding targets and underlying mechanisms has yet to be thoroughly investigated.

Reactive oxygen species (ROS), which are naturally produced as a by-product of aerobic respiration within cells, exhibit a dual nature in cellular biological processes, functioning both as essential signaling molecules and as potential mediators of cellular damage. For instance, hydrogen peroxide (H_2_O_2_) can function as the signalling molecule selectively modifying and regulating the function of many proteins, which thus supports cell behaviour. Elevated concentrations of ROS can result in harm and toxicity to cellular components such as DNA, proteins, and lipids [19]. Moreover, ROS-induced ER stress also critical for inducing cell death. To control ROS, cells adopt an intricate antioxidant system such as superoxide dismutases (SODs), catalase (CAT), peroxiredoxins (PRDXs) and glutathione peroxidases (GPXs). PRDXs abundant thiol-dependent peroxidases that convert H_2_O_2_ to water using thioredoxin (TRX) and NADPH [20, 21]. Expressions of most PRDX proteins in various cancers are increased and may provide new targets for treatment.

ER stress and autophagy are two important processes that regulate cell homeostasis. Unfolded or misfolded proteins accumulated in the ER induces unfolded protein respons (UPRs) to ensure the authenticity and integrity of proteins. The heat shock protein family A (Hsp70) member 5 (BIP; GRP78), which is found in the lumen of the endoplasmic reticulum (ER), serves as a key regulator. It accomplishes this through its interaction with three transmembrane proteins situated in the endoplasmic reticulum (ER): ATF6 (activating transcription factor 6), IRE1 (inositol-requiring enzyme 1), and PERK (protein kinase R-like endoplasmic reticulum kinase) [22, 23]. Autophagy is a cellular mechanism that promotes the breakdown and recycling of cellular components. Autophagy exhibits a dualistic role in oncogenesis that is strictly dependent on tumor stage and microenvironmental context. During tumor initiation, this lysosomal degradation pathway serves as a critical tumor suppressor mechanism by eliminating potentially oncogenic protein aggregates and dysfunctional mitochondria through selective autophagy (e.g., mitophagy), thereby preserving genomic integrity. Paradoxically, established tumors co-opt autophagic flux to sustain their metabolic demands during therapeutic challenge and metastatic dissemination, particularly under hypoxic and nutrient-deprived conditions characteristic of the tumor niche [24]. The interplay between ER stress signaling and autophagy constitutes a critical regulatory axis determining cellular fate, with both pathways demonstrating significant involvement in cell death mechanisms. However, whether and how of these two processes are involved in GA-induced cell death are unclear.

Activity-based protein profiling (ABPP) is increasingly being recognized as a powerful approach that combines activity-based probes with proteomics technologies, enabling the investigation of the protein targets of small bioactive molecules [25, 26]. Small molecules that are modified by probes maintain their pharmacological function to covalently attach to targets, serving the probe as the reporter group. This approach has contributed to understanding the pharmacology and side effects of various bioactive compounds, including curcumin, and aspirin [27, 28].

In this study, by using clear cell renal cell carcinoma as a cell model, we explored the mechanisms through which GA induce cell death. By integrating proteomics, ABPP study, and cellular and molecular studies, we reveal that GA covalently binds to PRDX1 and suppresses its activity, which leads to an increase in ROS levels and subsequent modulating ER-stress and autophagy for apoptotic cell death. This revelation illuminates the fundamental mechanism of GA's antitumor activity and underscores its potential as a potent anticancer drug, paving the way for innovative advancements in cancer therapy.

## Materials and methods

### Cell culture

Human renal clear cell adenocarcinoma cell line 786-O (CL-0010) was purchased from Wuhan Prosperity Life Science and Technology Co Ltd (Wuhan, China). Cells were kept at 37 °C with 5% CO_2_ in an incubator and grown in RPMI-1640 medium (Gibco, 11875093) enriched with 10% fetal bovine serum (ExCell, 16140071) and 100 U/mL of penicillin–streptomycin. The cells were passaged at intervals when they reached 80–90% confluence.

### Reagents

Cell viability kit-8 (CCK-8) (C0037) was obtained from Beyotime Biotech (Nanjing, China). 4-Phenylbutyric acid (4-PBA) (S3592), N-Acetylcysteine (NAC) (S1623) were purchased from SelleckChem (Houston, USA). Membrane Linker V and Propidium Iodide (PI) Apoptosis Detection Kit (KGA1013) was sourced from KeyGEN BioTECH (Nanjing, China). Gambogic acid (A0820) was purchased from Must Biotechnology (Chengdu, China). Torin1 (A8312), and Chloroquine (CQ) (S6999) were purchased from SelleckChem (Houston, USA). Reagents for click chemistry and LC–MS/MS analyses comprised the following: TAMRA azide (catalog number T10182), biotin azide, and Tris[(1-hydroxypropyl-1*H*-1,2,3-triazol-4-alkyl)methylamine] (THPTA), all obtained from ClickChemistryTools in the USA (product number 762342). Sodium ascorbate (NaVc) and copper sulfate (CuSO_4_) were acquired from Aldrich, a subsidiary of Sigma-USA. High-capacity neutral Streptavidin agarose beads (SA004100), triethylammonium bicarbonate (TEAB), and sequencing-grade modified trypsin were sourced from Thermo USA. Additionally, the PierceTM BCA Protein assay kit (catalog number 23225) was used for protein quantification, also from Thermo Fisher Scientific, USA. The ROS indicator, 2′,7′-dichlorodihydrofluorescein diacetate (DCFH-DA), with the product code E004-1-1, was purchased from Beyotime Biotech in Nanjing, China. Iodoacetamide-yne (IAA-yne) (I10189) from Thermo fisher (Massachusetts, USA). Rabbit anti-mouse antibody Bcl-2 (60178-1-lg), rabbit anti-mouse antibody BIP (11587-1-AP), rabbit anti-mouse antibody PRDX1 (15816-1-AP), rabbit anti-mouse antibody CHOP (15204-1-AP) Proteintech (Wuhan, China). Rabbit anti-mouse antibody Cle-caspase 3 (9664), mouse anti-mouse antibody GAPDH (2118S), rabbit anti-mouse antibody ATF4 (11815S), rabbit anti-mouse antibody eIF2α (5324S) and rabbit anti-mouse antibody p-eIF2α (9721S) were purchased from Cell Signaling Technology (Massachusetts, USA). Sheep anti-rabbit IgG (A0208) and sheep anti-mouse IgG (A0192), were purchased from Biotech (Nanjing, China).

### Cell viability assay

Plate 5 × 10^3^/well cells in a 96-well plate and then incubate with indicated concentrations of GA overnight for 24 h, then add 10 μL/well CCK-8, 37 °C for 1 h, and measure the absorbance at 450 nm wavelength.

### Apoptosis assay

Cells plated at 2 × 10^3^/well, incubated overnight for stable growth, ready for apoptosis eval. Following this, the cells were treated with GA for 24 h and subsequently stained with FITC-conjugated Annexin V along with PI, in accordance with the manufacturer’s guidelines. The data were gathered utilizing a CytoFLEX flow cytometer (Beckman) and analyzed thereafter using FlowJo v10 software.

### Western blot

Proteins from each cellular group were extracted utilizing a 1× SDS lysis buffer, followed by sample preparation after assessing the protein concentration with a BCA kit. Samples loaded at ≥20 μg/well, SDS-PAGE, proteins transferred to 0.2 μm PVDF (Merck), blocked with 5% milk (1 h), incubated with primary abs (4 ℃, overnight) including GAPDH, BIP, P62, LC3B, Cle-caspase 3, Bcl-2, ATF4, CHOP, eIF2α, and p-eIF2α on a shaking table. Immunoreactive bands were made visible by incubating with HRP-conjugated secondary antibody, then applying ECL reagent (Bio-Rad). The quantification and normalization of protein band intensities in Western blots were done using Image J (USA).

### In situ fluorescent labeling experiments

Cells grown in 6-well plates to 80–85% confluency, ready for further experiments/harvesting. After being treated with IAA—Probe (10 μM) or GA (20 μM) for 1 h, the cells were washed with pre-chilled PBS. Protein was extracted by using RIPA lysis buffer which was supplemented with a protease inhibitor cocktail. An equal volume of the lysate was then mixed with click chemistry reagents, consisting of 1 mM NaVc, 100 μM THPTA, 1 mM CuSO_4_, and 50 μM TAMRA-azide, and incubated for a duration of 2 h. Proteins precipitated with cold acetone, resuspended in buffer, separated by SDS-PAGE, stained with CBB, and analyzed by laser scanner (Azure Sapphire). To achieve fluorescent labeling (FL) of the recombinant proteins, protein (0.5–1 μg/μL) incubated with probe in PBS for 1 h, then click reaction. SDS-PAGE separation and visualization as before. For competitive labeling experiments, pretreatment with agents for 30–120 min before FL procedure.

### Pull down and LC-MS/MS based targets identification

Cells treated with GA (100 μM, 1 h), then with competitive agents (1 h). Extracted proteins underwent click reaction, dissolved in 1.5% SDS, and diluted to 0.1% SDS with PBS. Supernatant incubated with streptavidin beads (4 h, RT), washed (1% SDS, 0.1% SDS^1^ note: typo likely, use consistent SDS conc.), pulled-down proteins enriched, separated by SDS-PAGE, detected by WB [29, 30]. For LC-MS/MS: samples reduced/alkylated (DTT/iodoacetamide), digested (trypsin), labeled (TMT), analyzed (LC-MS/MS, Thermo).

### Colony formation assay

Cells seeded in 6-well plates (1 × 10^3^ cells/well), cultured with GA (250, 500, 750 nM) for 24 h, then in normal medium (7 days). Colony growth assessed by staining with 0.5% crystal violet, enabling quantitative/qualitative evaluation of gambogic acid's effect on cell proliferation. Colonies were defined as clusters of at least 50 cells.

### Cellular thermal shift assay (CETSA)-Western blot (WB)

786-O cells were lysed and incubated with GA (100 μM) at room temperature for 1 h. Subsequently, the lysate was divided into test tubes and heated to temperatures ranging from 50 to 75 °C. Following centrifugation, the soluble supernatants were mixed with a loading buffer and analyzed by western blot.

### Expression and purification of PRDX1

The plasmid, which was constructed correctly, was introduced into an expression strain (BL21, Transcetta) for the purposes of expression and purification. Post-induction, the cells were re-suspended in a binding buffer composed of 20 mM Tris–HCl (pH 8.0) and 200 mM NaCl. They were then disrupted using sonication while being maintained on ice. Following sonication, the supernatant was isolated and collected. The supernatant obtained was subsequently applied to a Ni–NTA resin column from Qiagen, USA, pre-equilibrated with a buffer comprising 200 mM NaCl, 20 mM Tris–HCl, and imidazole at concentrations of either 20 or 50 mM, all adjusted to a pH of 8.0 for the washing process.The recombinant protein was eluted using a buffer comprising 200 mM NaCl, 20 mM Tris–HCl (pH 8.0), and 200 mM imidazole. Protein concentration is measured using the BCA kit, SDS-PAGE, and CBB staining [32].

### Cell transfection

Transient transfection was performed following the manufacturer's guidelines using Lipofectamine™ 3000 (L3000150, Invitrogen). To assess the formation of autophagosomes and autophagic flux, GFP-LC3 and RFP-GFP-LC3 plasmids were utilized as previously described [33, 34]. The shRNA targeting PRDX1 was inserted into the GL407 plasmid, and subsequently, the PRDX1 cDNA was introduced into the GL124 plasmid for the purpose of overexpression (OE). Cells were fixed with 4% paraformaldehyde (PFA) to maintain their structural integrity after being stained with crystal violet. DAPI was used to stain the nuclei for observation. Images were captured using a Nikon ECLIPSE Ti2 microscope, enabling analysis of nuclear morphology, colony size, and cell distribution to understand Gambogic acid's effects on proliferation. The captured images were analyzed using ImageJ software to ascertain the number of GFP-LC3 puncta per cell.

### Determination of mitochondrial membrane potential

Cells seeded in 6-well plate at 1.5 × 10^5^/well, cultured overnight. Incubated with GA (250, 500, 750 nM) for 24 h. Trypsinized, resuspended in JC-1 solution, stained for 20 min at 37 °C, 5% CO_2_. Resuspended in PBS for flow cytometry to analyze GA effects on mitochondrial function and cellular viability.

### Detection of ROS generation in cells

Cells seeded in 6-well plate (1.5 × 10^5^/well), incubated overnight, then treated with GA (250, 500, 750 nM) for 16 h. Collect the cells and stain them with ROS staining working solution at 37 °C for 1 h (turn and mix every 10 min to ensure full contact between the probe and the cells). After staining, centrifuge and discard the supernatant, and wash twice with PBS. After being resuspended in 1 mL of PBS, the cells were prepared for flow cytometry analysis to quantify the production of reactive oxygen species (ROS), which was assessed based on the fluorescence intensity of DCFH-DA.

### Proteomics and GO analysis

786-O cells were exposed to 750 nM GA for 24 h. Subsequently, the cells were harvested and proteins extracted using a 1% sodium deoxycholate (SDC) buffer with 8 M urea. Protein samples are reduced by DTT. IAA alkylation stabilizes thiol groups. Trypsin digestion yields peptides for MS. Desalting removes impurities. Desalted peptides are analyzed by LC-MS/MS in DIA mode. This process enabled comprehensive analysis of all peptides in the sample for protein identification and quantification. Throughout the experiment, differential proteins were identified based on absolute fold changes ≥1 and *P*-values (FDR) < 0.05 between the control and GA-treated groups. Subsequently, a volcano plot was generated using the Bioadder website (https://www.bioladder.cn). Following analysis of the differential proteins with the DAVID + version, we chose to visualize the functional map focusing on biological processes and KEGG pathway enrichment. (https://david.ncifcrf.gov/).

### Data analysis

Data presented as mean ± SEM (indicator of sample mean variability). One-way ANOVA evaluates variations among multiple groups, while *t*-tests compare statistical significance between two groups. Insights from statistical comparisons enabled informed interpretation of results. The analysis utilized GraphPad Prism 8.0 software (San Diego, CA, USA), denoting significant results with asterisks (*P < 0.05, **P < 0.01, ***P < 0.001).

## Results

### GA induced apoptosis in renal carcinoma cells

**Fig. 1:**
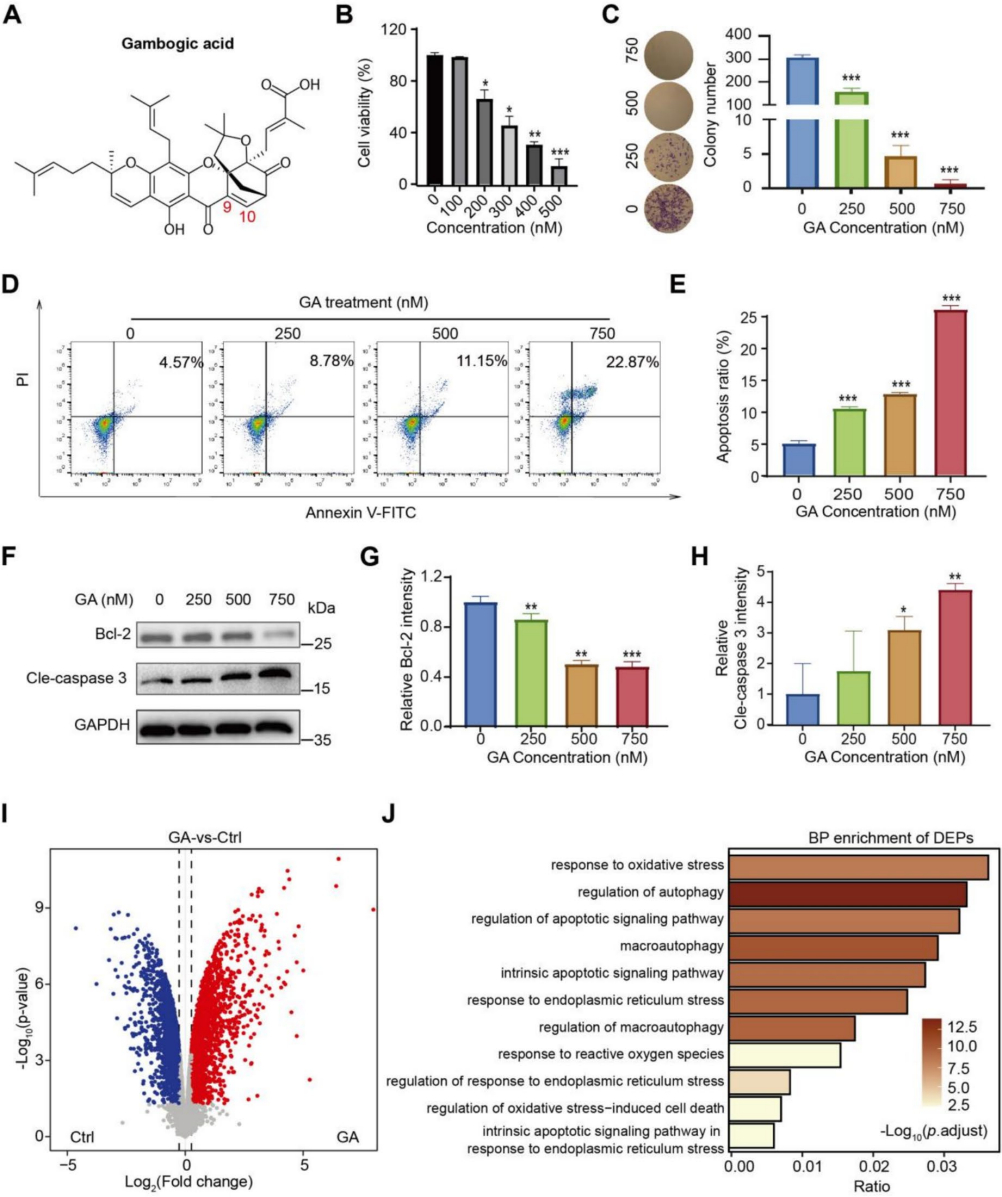
Cytotoxicity of GA towards 786-O cells. **A** Molecular structure of GA. **B** CCK-8 assay showed GA's cytotoxic effects after 24 h treatment. **C** 786-O cells with specified concentrations of GA for 24 h, followed by staining with crystal violet. After 24 h GA treatment, 786-O cells exhibited characteristic apoptotic morphology by flow cytometry (**D**), with apoptotic indices quantified in **E** showing concentration-dependent increases. The expression of apoptosis markers (Bcl-2, Cle-caspase 3) was detected by western blotting after 786-O cells were treated with indicated concentrations of GA for 24 h (**F**) and quantitatively analyzed (**G**, **H**). **I** Volcano plot of differential expressed proteins after GA treated for 24 h in 786-O cells (n = 3). **J** Analysis of the enrichment of upregulated proteins subsequent to GA treatment. Data (means ± SEM, ≥3 exp.) showed significant differences vs. control (*P < 0.05, **P < 0.01, ***P < 0.001)

GA is a naturally occurring xanthonoid with a broad range of anticancer activities (Fig. 1A). In order to determine the cytotoxicity of GA towards renal carcinoma cells, CCK-8 assay was applied. 786-O cell viability decreased dose-dependently with GA treatment for 24 h (Fig. 1B). The IC50 value for GA was 266.6 nM. The cytotoxic effects of GA in 786-O cells were further validated by colony formation experiments, showing a dose-dependent decrease in clone number (Fig. 1C). In addition, Annexin-V FITC/PI assay showed increased cell death with higher GA concentrations, indicating late apoptosis/necrosis and cytotoxicity in 786-O cells (Fig. 1D, E), indicating the pro-apoptotic effect of GA. To verify GA-induced apoptosis in 786-O cells, the apoptosis negative regulator Bcl-2 and pro-apoptosis protein Cle-caspase 3 were detected by western blot. As shown in Fig. 1F–H, after GA treatment for 24 h, up-regulated Cle-caspase 3 and down-regulated Bcl-2 were observed in 786-O cells, demonstrating GA may trigger mitochondrial apoptosis pathway of kidney cancer cells. To further reveal the mechanism underlying GA-induced cell apoptosis, comparative proteomics analysis was performed, obtaining 1952 up-regulated proteins and 2172 down-regulated proteins (Fig. 1I). Go analysis of differentially expressed proteins (DEPs) showed endoplasmic reticulum stress, autophagy, and endogenous apoptosis were significantly enriched (Fig. 1J).

### GA-induced apoptosis was associated with accumulation of ROS

**Fig. 2:**
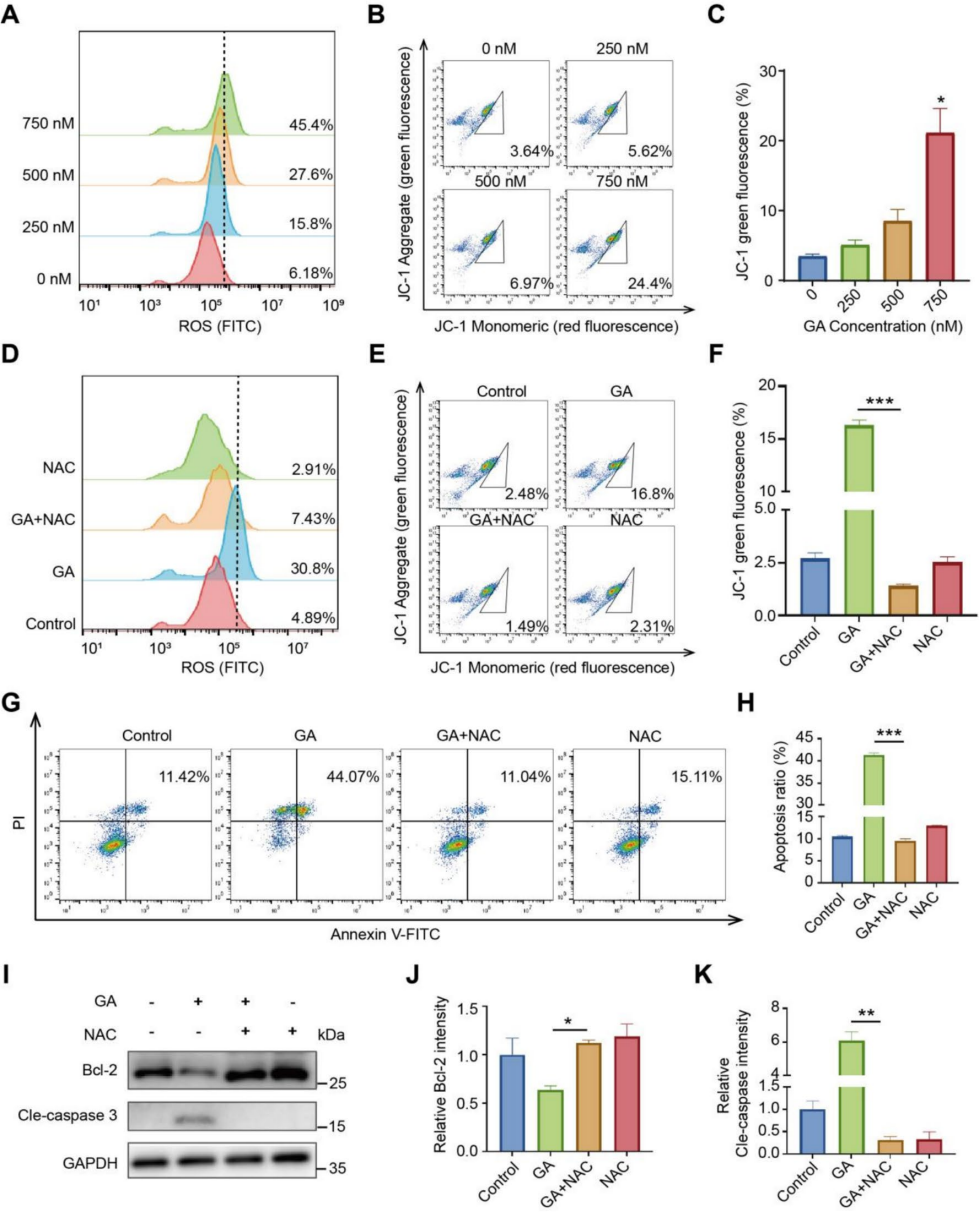
GA promotes apoptosis in renal cancer cells through elevating ROS levels. After 786-O cells were exposed to specific concentrations of GA for 24 h, ROS levels were detected by DCFH-DA staining and followed by flow cytometer (**A**). **B**–**C** Cells treated with GA for 24 h, stained with JC-1, analyzed by flow cytometry for mitochondrial potential. ROS inhibitor NAC reverse GA-induced ROS build-up (**D**) and the decline in mitochondrial membrane potential (**E**, **F**). **G**, **H** NAC can restore GA-induced apoptosis. **I**–**K** 786-O cells pretreated with/without 2 mM NAC for 1 h, then exposed to GA for 24 h, apoptosis markers were detected by western blotting. Data (mean ± SEM, ≥3 experiments) indicate significant differences in comparison to controls (*P < 0.05, **P < 0.01, ***P < 0.001)

Given the significant enrichment of ‘response oxidative stress’ and ‘regulation of oxidative stress-induced cell death’ of our proteomics data upon GA treatment (Fig. 1J), we speculated GA-induced apoptosis may be attributed to excessive oxidative stress. So, we detected levels of intracellular ROS and mitochondrial transmembrane potential after GA treatment first. GA treatment dose-dependently increased ROS and decreased mitochondrial potential in 786-O cells (Fig. 2A–C). NAC, a ROS scavenger, rescued these effects (Fig. 2D–F), indicating ROS's role in GA-induced mitochondrial apoptosis in 786-O cells. Then effects of NAC on GA-induced apoptosis was detected by flow cytometry, showing NAC completely diminished cell death (Fig. 2G, H). These results were further demonstrated by the restore of Bcl-2 and Cle-caspase 3 levels upon GA treatment (Fig. 2I–K). Taken together, the accumulation of intracellular ROS accounted for GA-induced mitochondrial apoptosis.

### GA-induced cell death via ROS-mediated ER stress

**Fig. 3:**
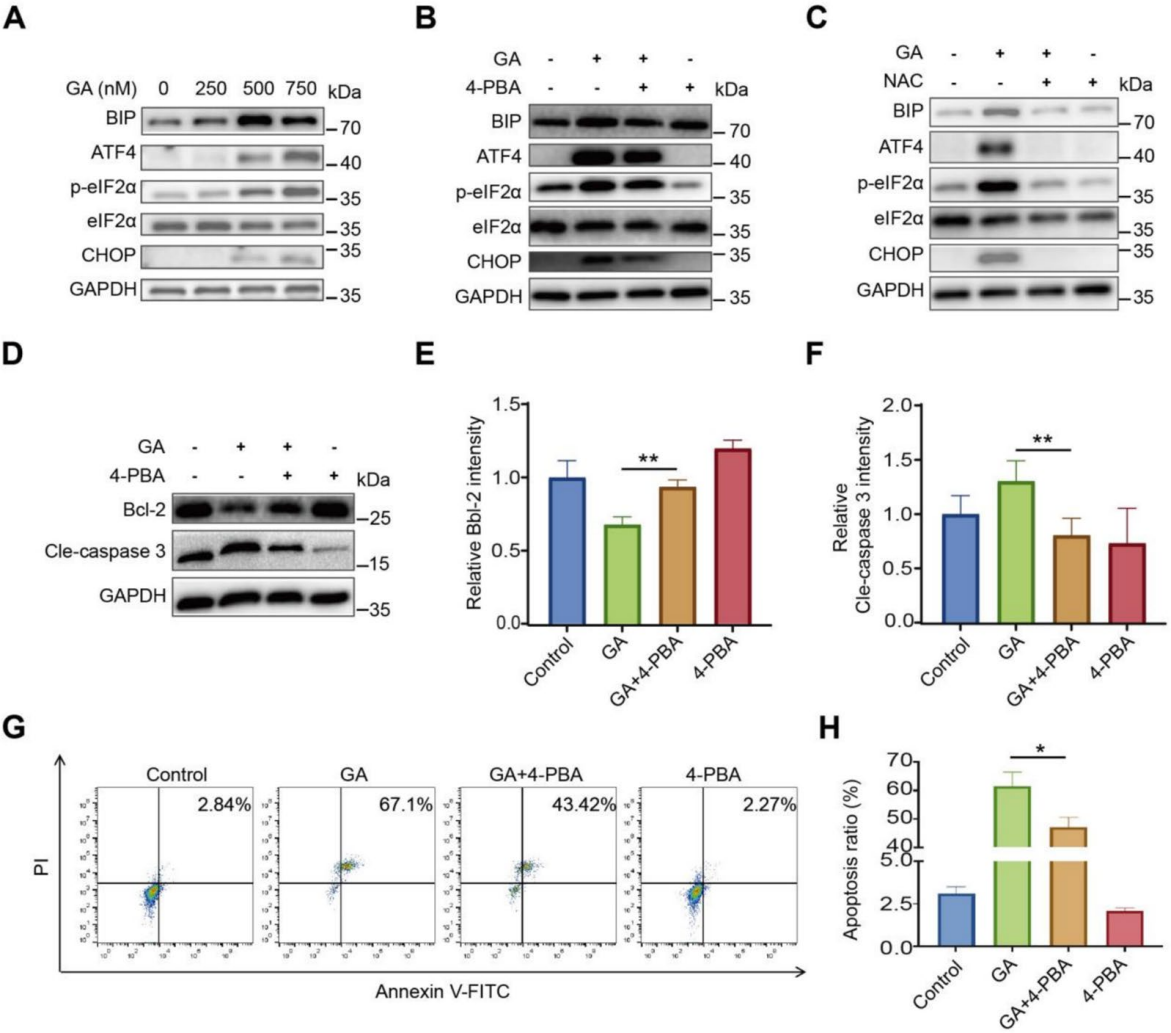
GA initiates ER stress-mediated apoptosis by elevating ROS. **A** GA induces ER stress marker expression in 786-O cells treated with various concentrations of GA. **B** Prior to exposure to GA for 24 h, 786-O cells were pretreated with 1 mM of 4-PBA for 1 h, and subsequently, the samples underwent western blotting analysis. **C** 786-O cells were pretreated with 2 mM of NAC for 1 h before being exposed to GA for 24 h, followed by western blotting analysis of the samples. **D**–**H** After 4-PBA (1 mM, 1 h) pretreatment, 786-O cells were exposed to the indicated concentrations of GA for 24 h, western blotting was performed to analyze Cle-caspase 3 and Bcl-2 (**D**, **E**), and cell apoptosis was detected by flow cytometry **G**–**H**. Data (mean ± SEM, ≥3 independent experiments) show significant differences compared to controls (*P < 0.05, **P < 0.01, ***P < 0.001)

Accumulated ROS was reported to cause ER stress which was also enriched in GO analysis in GA treated (Fig. 1J). Activation of ER stress was reported to induce mitochondrial dysfunction and cell apoptosis [35]. Thus, we detected the protein levels of BIP-eIF2α-ATF4-CHOP axis, a canonical ER stress signaling pathway in GA treated cells. As shown in Fig. 3A, after GA treatment, the expression of BIP, p-eIF2α, ATF4 and CHOP were significantly up-regulated in a does-dependent manner. While the increase of these ER stress-related proteins was diminished effectively by ER Stress inhibitor 4-PBA, indicating that GA activated endoplasmic reticulum stress pathway (Fig. 3B). To elucidate the effects of ROS on GA-induced ER stress, these proteins were detected after NAC co-treatment, western blot results showed the increased eIF2α, GRP78, ATF4, and CHOP were efficiently restored to normal levels in the presence of ROS scavenger NAC, suggesting that ROS was responsible for the endoplasmic reticulum stress activated by GA exposure (Fig. 3C). Then we determined the impact of GA-induced ER stress on cell apoptosis. 4-PBA dramatically reversed the decreased level of Bcl-2 and the elevated expression of cle-caspase3 due to GA treatment (Fig. 3D–F), implying that ER stress contributed to GA-triggered apoptosis of 786-O. It was further supported by flow cytometry results, showing 4-PBA remarkable attenuated GA-triggered apoptosis (Fig. 3G, H). In summary, GA triggered endoplasmic reticulum stress through the excessive ROS production and ultimately led to cell apoptosis.

### GA induces autophagy in renal cancer cells

**Fig. 4:**
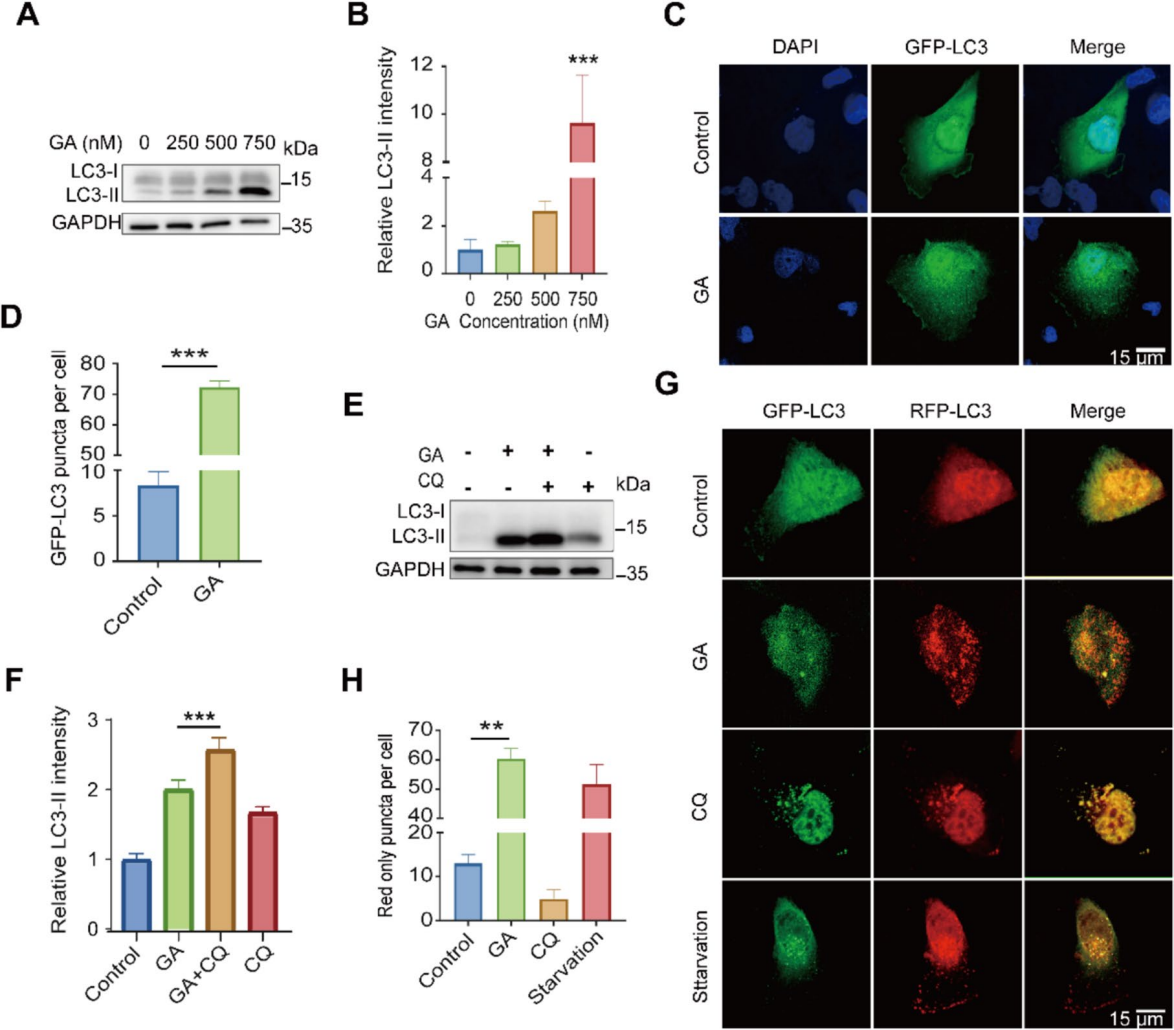
GA promotes autophagic flux in renal cancer cells. **A**, **B** After treating with GA at specific concentrations for 24 h, the relative expression level of the autophagy marker LC3-II was assessed using western blotting (**A**). Quantitative analysis of LC3-II (**B**). **C**, **D** Following the treatment of 786-O cells transiently expressing GFP-LC3 with GA for 24 h, samples were gathered and analyzed using immunofluorescence microscopy (**C**) and a quantitative assessment of the number of GFP-LC3 dots per cell was conducted (**D**). **E**, **F** After treating cells with CQ alone or in combination with GA for 24 h, the relative expression level of the autophagy marker LC3-II was determined through western blotting and subjected to quantitative analysis. **G**, **H** RFP-GFP-LC3-transfected 786-O cells treated with GA for 24 h were analyzed using immunofluorescence microscopy. Starvation served as the positive control, while the lysosomal inhibitor CQ acted as the negative control. Data (shown as mean ± SEM from at least three experiments) exhibit significant differences in comparison with controls (*P < 0.05, **P < 0.01, ***P < 0.001)

In view of significant enrichment of ‘autophagy’ in DEPs upon GA treatment (Fig. 1J), we determined whether GA induces autophagy. Through the analysis of the transformation from LC3-I to LC3-II, our findings indicated that GA elevated LC3-II levels in 786-O cells in a dose-responsive manner. (Fig. 4A, B). The increase of LC3 in response to GA treatment was also evidenced by the number of LC3 puncta in 786-O cells expressing GFP-LC3 plasmids (Fig. 4C, D). To dissect the increase of LC3-II was owing to the inhibition or initiation of autophagic flux, 786-O cells were simultaneously exposed to GA and CQ, an autophagy inhibitor by inhibiting the fusion of autophagosome and lysosome. As shown in Fig. 4E, F, LC3-II levels were further upregulated in the co-presence of CQ and GA comparing with CQ or GA alone, indicating GA activated autophagic flux. Furthermore, we assessed the autophagic flux status by utilizing the mRFP-GFP-LC3 plasmid. As a positive control, starvation activated autophagy, indicated by increased autolysosomes (red onlypuncta), while autophagy inhibitor CQ markedly reduced autolysosomes and increased autophagosomes (yellow puncta). GA treatment resulted in an increase in the number of red puncta (Fig. 4G, H), providing further evidence of its role in enhancing autophagy flux.

### GA-triggered autophagy through ROS-mediated ER stress

**Fig. 5:**
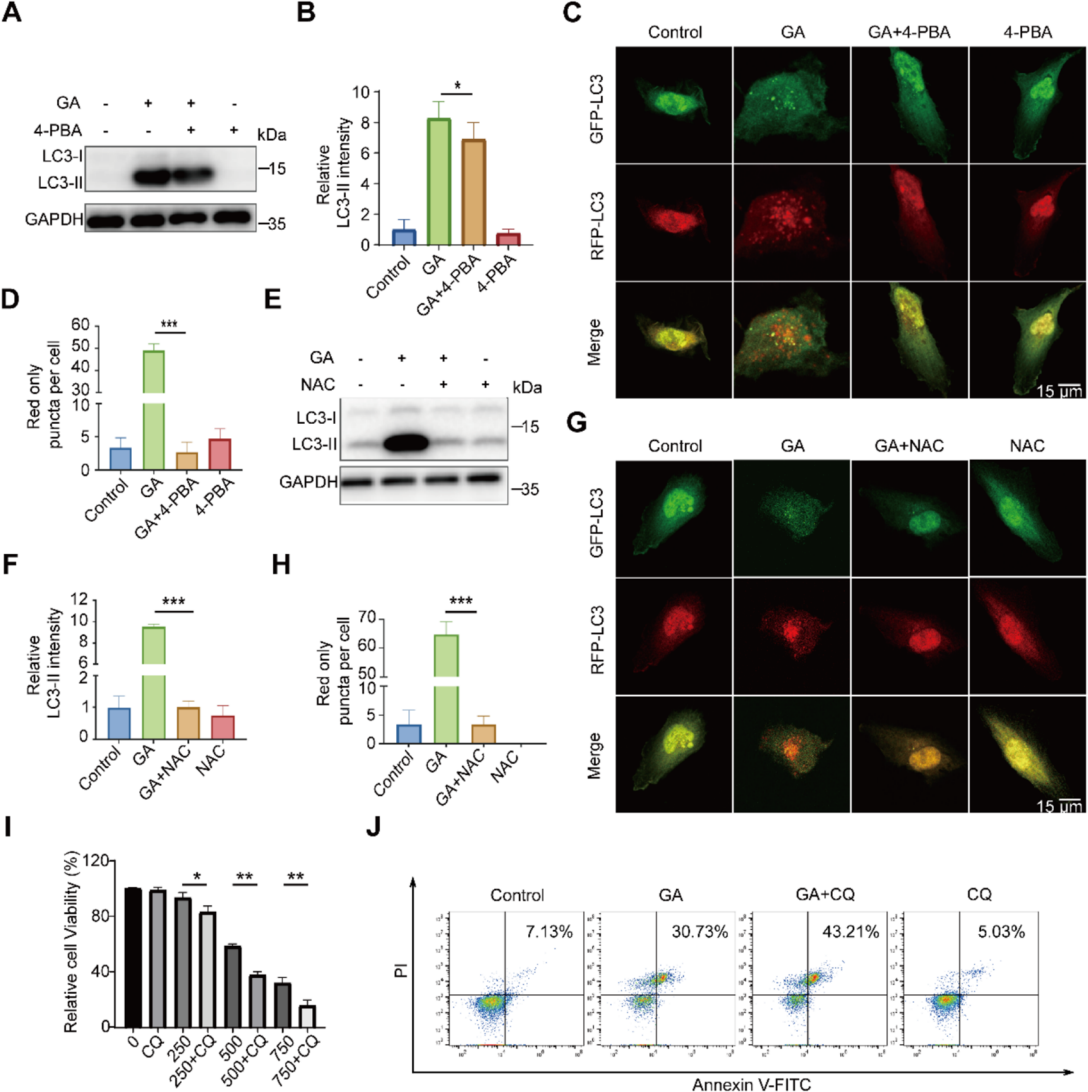
ROS-induced ER stress is contributed to GA-induced autophagy. **A**, **B** 786-O cells were pretreated with 1 mM 4-PBA for 1 h prior to 24-h GA exposure. Protein lysates were then subjected to western blot analysis, followed by densitometric quantification of band intensities. **C**, **D** 786-O cells transiently expressing RFP-GFP-LC3 plasmid were pre-treated with 4-PBA for 1 h and then treated with GA for 24 h. Autophagic flux was assessed through quantitative fluorescence microscopy analysis of LC3 puncta formation. **E, F** Pretreatment of 786-O cells with 2 mM NAC for 1 h was followed by 24 h GA exposure. Subsequent western blot analysis with densitometric quantification revealed dose-dependent alterations in protein expression profiles. **G**, **H** Following 24-h co-treatment with NAC and GA, 786-O cells transiently expressing the RFP-GFP-LC3 reporter construct were harvested and subjected to immunofluorescence microscopic analysis to assess autophagic flux. **I** CQ further promotes the decrease in cell viability induced by GA. **J** CQ leads to an increase in the apoptosis rate of GA-treated cells. Data (mean ± standard error of the mean (SEM), with no less than three experiments) display significant differences in comparison to controls (*P < 0.05, **P < 0.01, ***P < 0.001)

Cellular autophagy can be triggered in stress conditions including ER stress [36]. To define whether GA-induced autophagy was attributed to ROS-mediated ER stress activation in 786-O cells, LC3-II levels were measured after co-treatment of cells with ER stress inhibitor 4-PBA and GA. Western blot analysis revealed that 4-PBA mitigated the increase in LC3-II levels induced by GA (Fig. 5A, B), indicating ER stress caused autophagic response. Besides, the increased red only puncta by GA treatment was reversed to normal level by 4-PBA (Fig. 5C, D), indicating autophagic flux level was decreased by the inhibition of ER stress. Consistently, the suppression of ROS by NAC completely rescued GA-induced raises in LC3-II level and red puncta (Fig. 5E–H), indicating GA triggered autophagic response through ROS-induced ER stress.

Next, to ascertain the relationship between autophagy and cell death, we detected cell viability and death with or without co-treatment of the autophagy inhibitor CQ. CCK-8 assay results indicated that CQ potentiated the cytotoxicity of GA at each concentration tested (Fig. 5I), and FITC-Annexin V double staining also validated that inhibition of autophagy by CQ aggravated cell death induced by GA (Fig. 5J). Combined with the above findings, autophagy was an adaptive response to ROS-induced ER stress in response to GA treatment, which helped to avoid cell death.

### GA directly covalently targets PRDX1

**Fig. 6:**
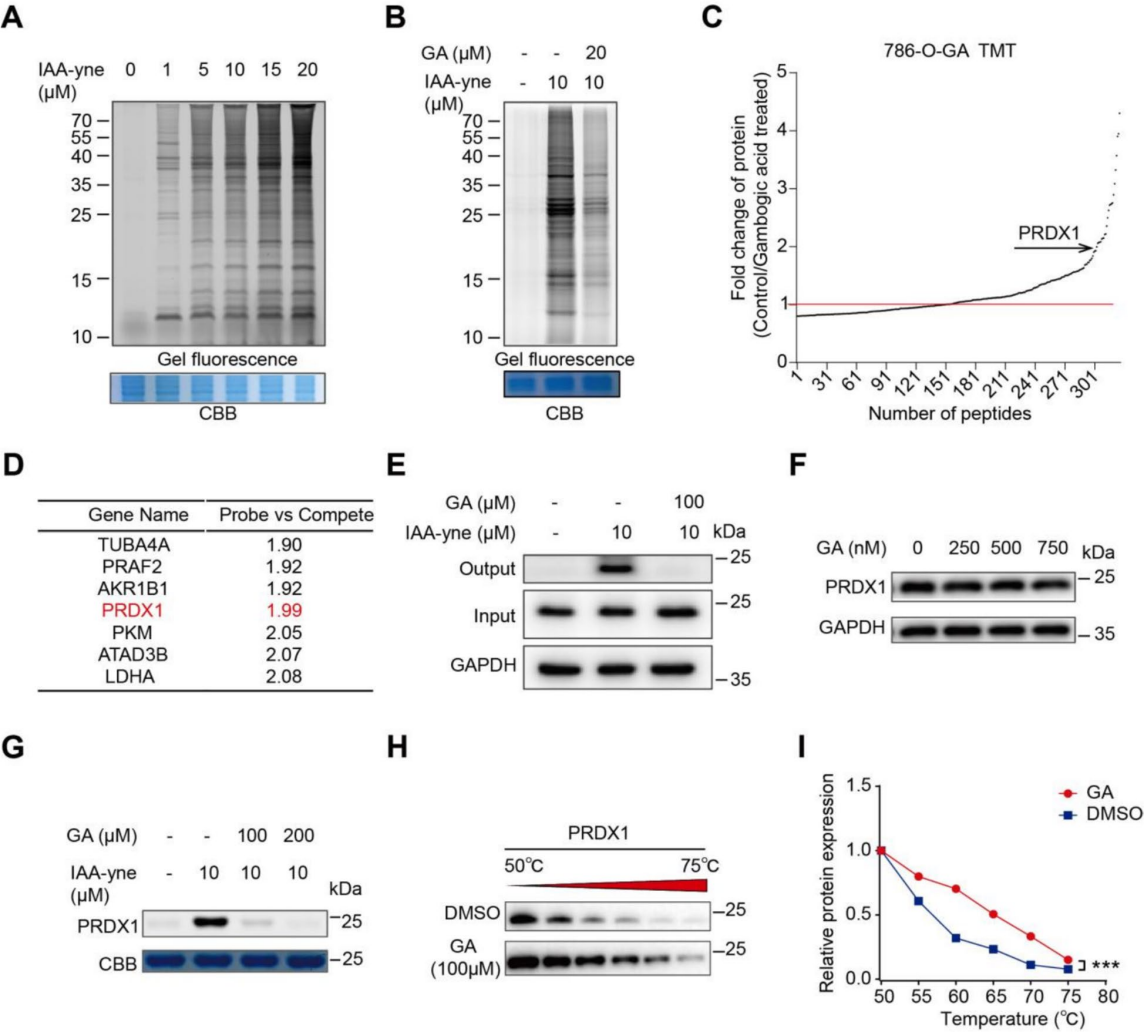
GA directly attaches to PRDX1. **A** In-situ protein labeling by IAA-yne happens in a dose-dependent fashion in 786-O cells. **B** GA contends with IAA-yne for binding to cysteine residues on proteins. **C** Chemical proteomics was employed to identify potential binding targets of GA in 786-O cells. **D** Catalogue of GA high-priority protein targets. **E** Verification of GA binding to PRDX1 via pull-down experiments. **F** GA treatment doesn't change PRDX1 protein expression. **G** Competitive binding between GA and IAA-yne. **H**, **I** CETSA—WB experiments indicate that PRDX1 has remarkable thermal stability in the presence of GA. Data (mean ± SEM, with no fewer than three experiments) indicate significant differences in comparison to controls (*P < 0.05, **P < 0.01, ***P < 0.001)

Considering GA is a classic Michael receptor owing to the α, β-unsaturated ketone substructure of GA at C10 (Fig. 1A) [37], we speculated the cytotoxicity of GA depended on covalent binding to cysteine-containing proteins. Therefore, we employed activity-based protein profiling (ABPP) technology to detect its binding protein targets within 786-O cells. First, IAA-yne probe was employed to alkylate cysteine-containing proteins in 786-O cells, and 10 μM IAA-yne probe was capable of alkylation, verified by the prominent protein bands (Fig. 6A). At a concentration of 20 μM, GA was able to significantly compete with the IAA-yne probe in labeling cellular proteins, as shown in Fig. 6B, suggesting that GA can form covalent bonds with cysteine residues of potential target proteins.

To identify the target proteins involved in GA-induced ROS redundancy, IAA-labeled proteomes with or with GA competition were enriched and quantified by TMT-based quantitative proteomics according to our previous protocols [38, 39]. We found that GA effectively competed with IAA-yne probe to react with cysteine-containing proteins (Fig. 6C). Among these candidate target, PRDX1 was particularly noticeable (Fig. 6D), mainly for two reasons: one is that it shows a large competitive effect, and the other is due to the physiological role of PRDX1 in redox regulation. Then we employed a pull-down experiment to verify GA disturbed the binding of IAA to PRDX1 in cell lysate, and our results confirmed that 100 μM GA treatment significantly eliminate the band of PRDX1 in the competition group (Fig. 6E). To investigate the reason behind the decreased interaction between PRDX1 and IAA in the presence of GA, we evaluated PRDX1 expression levels at varying GA concentrations. However, our findings demonstrated that GA had no impact on PRDX1 expression (Fig. 6F). This suggests that the diminished binding could be attributed to GA's competitive binding. Consequently, we purified recombinant human PRDX1 protein and incubated it with the IAA-yne probe, both in the presence and absence of GA. Our findings suggested that GA decreased the fluorescent labeling of PRDX1 by the IAA-yne probe, as illustrated in Fig. 6G, providing additional evidence that GA competes for binding with the cysteine-rich PRDX1. Additionally, a cellular thermal shift assay (CETSA) revealed that PRDX1 maintained significant thermal stability when exposed to GA (Fig. 6H, I), confirming that PRDX1 is a direct binding target of GA. In summary, the binding of GA to PRDX1 disrupts its functionality, potentially impacting the antioxidant and anti-apoptotic roles that PRDX1 plays in renal cancer cells.

### PRDX1 is responsible for GA-induced cell death

**Fig. 7:**
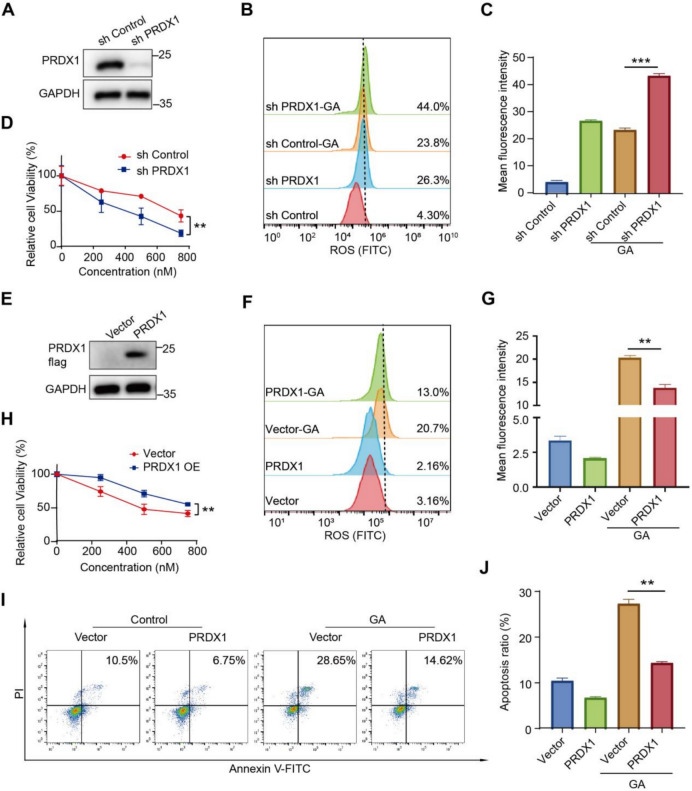
Role of PRDX1 in GA-induced apoptosis. **A** The protein expression levels of PRDX1 were determined by western blot analysis in cells transfected with either PRDX1-specific shRNA or control vector. **B**, **C** Reduction of PRDX1 expression promotes GA-induced ROS accumulation in 786-O cells. **D** PRDX1 knockdown enhances GA-induced inhibition of 786-O cell viability. **E** Over-expression of PRDX1 in 786-O cells. **F**, **G** Over-expression of PRDX1 restrains GA-induced ROS accumulation in 786-O cells. **H** Over-expression of PRDX1 weakens the inhibitory effect of GA on cell viability. **I**, **J** Over-expression of PRDX1 decreases the apoptosis rate of GA-treated 786-O cells. Data (means ± SEM, ≥3 exp.) showed significant differences vs. control (*P < 0.05, **P < 0.01, ***P < 0.001)

To verify GA-induced ROS disturbance relied on PRDX1, we knockdown (KD) the expression of PRDX1 by shRNA, and KD efficiency was verified by western blot (Fig. 7A). PRDX1 knockdown raised ROS levels, and GA treatment on PRDX1 KD 786-O cells further increased ROS levels comparing with GA alone, implying the role of PRDX1 in GA-caused ROS production (Fig. 7B, C). Furthermore, knocking down PRDX1 reduced the viability of 786-O cells treated with various concentrations of GA (Fig. 7D), demonstrating an enhanced cytotoxicity effect of GA.

In contrast, overexpression of PRDX1 alleviated GA-induced oxidative stress, evidenced by decreased ROS levels compared to GA-treated alone cells (Fig. 7G, H). In addition, overexpression of PRDX1 mitigates cell viability and cell death in GA treated cells (Fig. 7F–J), demonstrating critical role of PRDX1 in GA-induced cell death. Taken together, these results indicated that PRDX1 is critical for ROS production and subsequently cell death upon GA treatment.

## Discussion

**Fig. 8:**
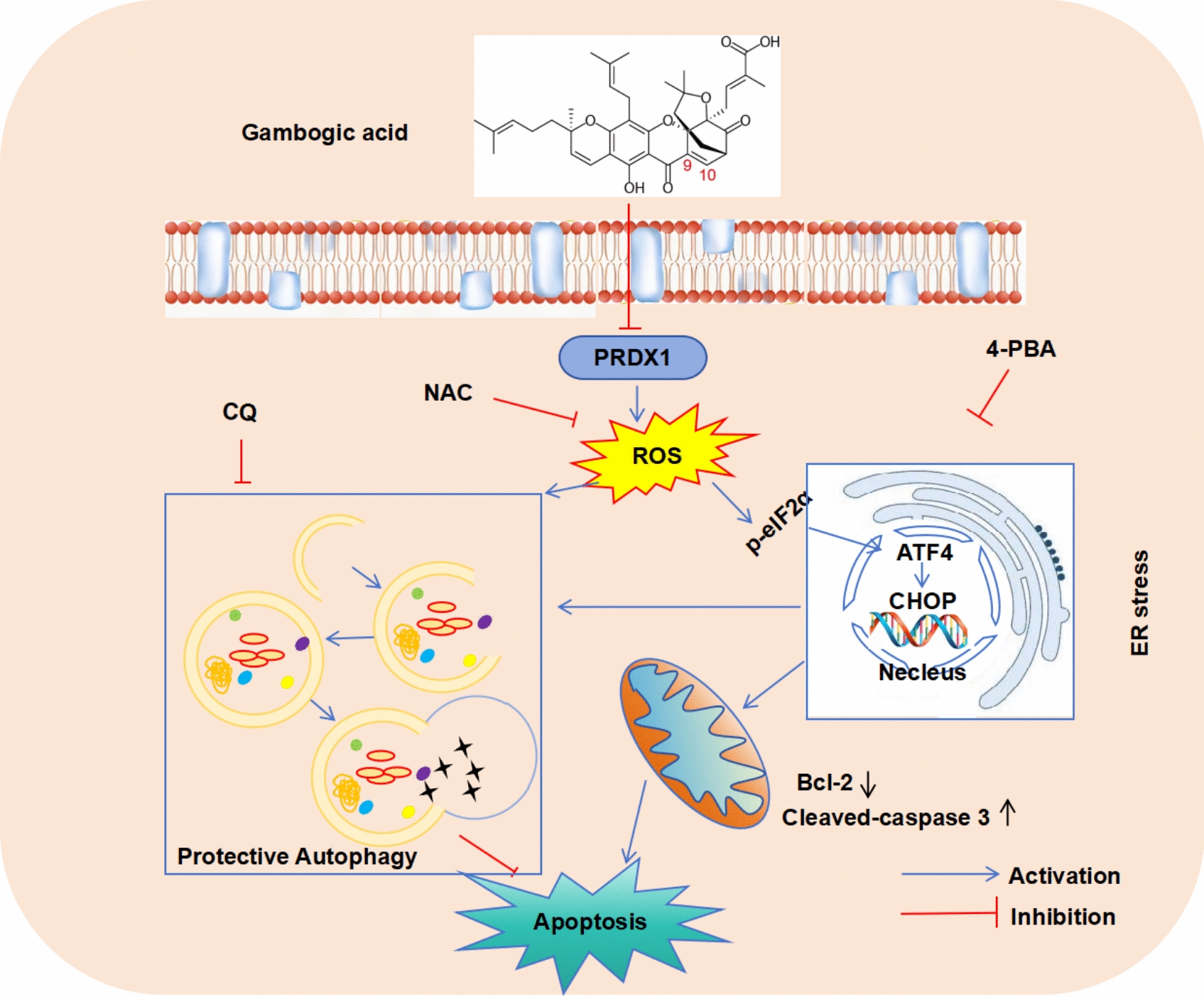
Diagrammatic illustration of the proposed mechanism underlying GA-induced toxicity. GA directly covalently binds to PRDX1, resulting in the accumulation of ROS. The accumulation of ROS, driven by the direct covalent binding of GA to PRDX1, further stimulates apoptosis through the activation of ER stress. Intriguingly, GA-induced ROS and the subsequent ER stress triggered by ROS also stimulate protective autophagy in cells

In this study, we found that GA showed significant cell toxicity against renal cancer cell 786-O cells. GA increased the generation of ROS by directly covalent binding to the antioxidant protein PRDX1, thereby triggering endoplasmic reticulum stress. In addition, the increase in ROS-mediated ER stress caused by GA treatment also initiated protective autophagy (Fig. 8). Our study discovered a new protein target of GA and provided new insights into our understanding molecular mechanisms for the cytotoxicity effects of GA.

Reactive oxygen species (ROS) like O2−, H_2_O_2_, ·OH are by-products of oxygen metabolism. It is generated through various pathways, including mitochondrial respiratory chain, NADPH oxidase, endoplasmic reticulum and peroxisomes. ROS are widely distributed throughout cells due to these various biosynthesis pathways, and they are essential for many physiological and pathological activities [37]. Previous research has demonstrated that GA can stimulate the production of ROS within various cancer cell lines. This elevation in reactive oxygen species subsequently triggers cellular apoptosis. Researchers have determined that in human esophageal cancer cells, GA inhibits cell proliferation, induces apoptosis, triggers cell cycle arrest, and disrupts mitochondrial membrane potential in a ROS-dependent manner [2].We discovered that GA concentration-dependently enhanced ROS formation in 786-O cells, which is in line with earlier observations. These results imply that the primary mechanism through which GA produces its harmful effects may be the production of ROS. Additionally, we found that ROS controlled the activation of autophagy, alterations in mitochondrial membrane potential, and elevated ROS formation in GA-treated renal cancer cells—all molecular processes that resulted in GA-induced apoptosis—while NAC therapy inhibited all these things happened.

Interestingly, autophagy, triggered by ROS, serves to eliminate damaged mitochondria and uphold cellular homeostasis, thus shielding cancer cells from the detrimental impacts of anticancer agents [40]. We found that when GA was used alone to treat renal cancer cells, GA induced autophagy, and this autophagy activation protects cells from apoptosis, whereas inhibition of autophagy can lead to apoptosis. Furthermore, the activation of autophagy by GA involves ROS-mediated ER stress, indicating a intricate interplay between these two pathways in response to GA treatment. However, how ER stress activates autophagy in the condition of GA treatment is still unclear. It is postulated that ATF4 may play a role because previous studies have shown that ATF4 controls the expression of multiple autophagy-related genes [41, 42].

To sustain a relatively balanced level of intracellular oxidation, cancer cells have developed a defense mechanism aimed at keeping reactive oxygen species (ROS) within physiologically acceptable limits. Certain antioxidant enzymes play a crucial role in neutralizing or transforming free radicals, thereby preventing excessive ROS from causing cellular damage [43]. Recently, the PRDX family has gained significant attention in anti-tumor investigations. These peroxiredoxins utilize thioredoxin as an essential intermediary electron donor to reduce ROS accumulation [44]. Among them, PRDX1 and PRDX2 are the two most studied members, exhibiting widespread and high expression in various tumors. We discovered that PRDX1 is a direct binding target of GA. GA inhibits the activity of PRDX1 by binding to PRDX1 and promotes the production of ROS, thereby inducing ER stress-mediated apoptosis. Furthermore, by PRDX1 overexpression and knockdown, we discovered that the apoptosis induced by GA in renal cancer cells can be significantly influenced. These results unveil a previously unrecognized protein target of GA and offer new perspectives for understanding the anti-cancer properties of GA.

Although our study has centered on PRDX1, it's noteworthy that other peroxiredoxin family members (particularly PRDX2) may also mediate GA's anticancer activity. Furthermore, emerging evidence suggests GA likely exerts its broad-spectrum antitumor effects through multi-target modulation, including but not limited to CNPY3 and BMI1, indicating a complex polypharmacological profile [45, 46]. Comprehensive mechanistic studies are needed to map the interaction networks between PRDX isoforms and GA's polypharmacological targets. The clinical relevance is highlighted by GA's ongoing Phase II evaluation [47] and PRDX1's emergence as a key mediator. Promising translational avenues include: (i) structure-based design of PRDX1 inhibitors, (ii) CRISPR-Cas9-mediated genetic validation, and (iii) development of PRDX1-based companion diagnostics. Such multidimensional research could transform RCC treatment paradigms through target-specific therapeutic stratification, positioning PRDX1 as a compelling focus for future investigation.

The anti-cancer effects of GA have been well-established [48, 49]. However, the molecular mechanism are unclear. In this manuscript, we mainly foucused on its molecular mechanism, especially tis direct binding targets. Here, for the first time as we known, we show PRDX1 is a direct binding target of GS. We found that GA induces cell death by inducing ER stress and modulating autophagy. GA-induced autophagy is involved in ER stress activation. Importantly, GA-induced ER stress and subsequently cell death is caused by increased ROS levels. Mechanistic studies show that PRDX1, a key antioxidant enzyme, is a directly covalent binding target of GA, and overexpression of PRDX1 mitigates GA-induced ROS production and subsequently cell death. The study identified PRDX1 is a potential directly covalent binding protein of gambogic acid, and elucidated its mechanism for inducing cell death that involving in ROS-mediated ER stress and autophagy regulation. The results obtained offer fresh perspectives on the mechanisms underlying the cytotoxic effects induced by gambogic acid, further suggesting that PRDX1 may represent a promising candidate for targeted therapy in the development of novel anticancer agents, notably for the treatment of kidney cancer.

The anti-cancer effects of GA are well established [50–52]. However, the molecular mechanisms are unclear. In this manuscript, we mainly focused on its molecular mechanism, especially tis direct binding targets. Notably, we found that GA-induced cell death with activation of prosurvival autophagy and lysosome inhibitor CQ further increase GA-induced toxicity, future studies to dissect the combination of GA and lysosome inhibitors for cancer treatment in vivo animal models is promising.

## Conclusion

In this investigation, by covalent binding to PRDX1, GA increases ROS levels, which leads to apoptosis mediated by endoplasmic reticulum stress, while also fostering cytoprotective autophagy in renal cancer cells. The findings of our study offer significant insights into the mechanisms responsible for GA's anticancer effects. In addition to offering fresh perspectives on the fundamental processes behind gambogic acid-induced cytotoxicity, these results imply that PRDX1 may be a viable therapeutic target for the creation of anti-cancer medications. Such as structure-based design of novel effective PRDX1 inhibitors and the combination of PRDX1 inhibitor with autophagy inhibitors for cancer treatment.

## Data Availability

The datasets used and/or analyzed during the current study are available from the corresponding author on reasonable request.

## Abbreviations

ABPP: activity-based protein profiling
CETSA: Cellular thermal shift assay
CQ: Chloroquine
DCFH-DA: 2',7'-dichlorodihydrofluorescein diacetate
DEPs: differentially expressed proteins
ER: endoplasmic reticulum
GA: Gambogic acid
IAA-yne: Iodoacetamide-yne
KD: knockdown
PERK: protein kinase R-like endoplasmic reticulum kinase
PI: Propidium Iodide
PRDX1: peroxiredoxin-1
RCC: Renal cell carcinoma
ROS: Reactive oxygen species
UPR: unfolded protein response

## Footnotes

1 Note: Typically, SDS concentration would be consistent (e.g., 1% SDS) [31].

## References

[1] Hatami E, et al. Gambogic acid: a shining natural compound to nanomedicine for cancer therapeutics. Biochim Biophys Acta Rev Cancer. 2020;1874(1): 188381. 32492470 10.1016/j.bbcan.2020.188381 PMC7484097

[2] Liu J, et al. Nanoscale features of gambogic acid induced ROS-dependent apoptosis in esophageal cancer cells imaged by atomic force microscopy. Scanning. 2022;2022:1422185. 35937670 10.1155/2022/1422185 PMC9337977

[3] Xia Z, Tang Z. Network pharmacology analysis and experimental pharmacology study explore the mechanism of gambogic acid against endometrial cancer. ACS Omega. 2021;6(16):10944–52. 34056247 10.1021/acsomega.1c00696 PMC8153951

[4] Yang Z, et al. Anti-angiogenesis in colorectal cancer therapy. Cancer Sci. 2024;115(3):734–51. 38233340 10.1111/cas.16063 PMC10921012

[5] Lan J, et al. Unlocking the anticancer activity of gambogic acid: a shift towards ferroptosis via a GSH/Trx dual antioxidant system. Free Radic Biol Med. 2024;218:26–40. 38570172 10.1016/j.freeradbiomed.2024.03.023

[6] Ren T, et al. Gambogic acid suppresses nasopharyngeal carcinoma via rewiring molecular network of cancer malignancy and immunosurveillance. Biomed Pharmacother. 2022;150: 113012. 35658246 10.1016/j.biopha.2022.113012

[7] Da M, et al. Therapeutic effect and metabolic fingerprinting of triple-negative breast cancer cells following exposure to a novel pH-responsive, gambogic acid-loaded micelle. Nanotechnology. 2023;35(11):115101. 10.1088/1361-6528/ad1448. 10.1088/1361-6528/ad1448 38081078

[8] Wang M, et al. Gambogenic acid inhibits basal autophagy of drug-resistant hepatoma cells and improves its sensitivity to adriamycin. Biol Pharm Bull. 2022;45(1):63–70. 34980780 10.1248/bpb.b21-00511

[9] Zhang Q, et al. Gambogic acid exhibits promising anticancer activity by inhibiting the pentose phosphate pathway in lung cancer mouse model. Phytomedicine. 2024;129: 155657. 38692076 10.1016/j.phymed.2024.155657

[10] Bahadoram S, et al. Renal cell carcinoma: an overview of the epidemiology, diagnosis, and treatment. G Ital Nefrol. 2022;39(3):2022. 35819037

[11] Yang J, Wang K, Yang Z. Treatment strategies for clear cell renal cell carcinoma: past, present and future. Front Oncol. 2023;13:1133832. 37025584 10.3389/fonc.2023.1133832 PMC10070676

[12] Shi X, et al. Gambogic acid induces apoptosis in diffuse large B-cell lymphoma cells via inducing proteasome inhibition. Sci Rep. 2015;5:9694. 25853502 10.1038/srep09694 PMC4894437

[13] Duan D, et al. Gambogic acid induces apoptosis in hepatocellular carcinoma SMMC-7721 cells by targeting cytosolic thioredoxin reductase. Free Radic Biol Med. 2014;69:15–25. 24407164 10.1016/j.freeradbiomed.2013.12.027

[14] Yang J, et al. Gambogic acid deactivates cytosolic and mitochondrial thioredoxins by covalent binding to the functional domain. J Nat Prod. 2012;75(6):1108–16. 22663155 10.1021/np300118c

[15] Palempalli UD, et al. Gambogic acid covalently modifies IkappaB kinase-beta subunit to mediate suppression of lipopolysaccharide-induced activation of NF-kappaB in macrophages. Biochem J. 2009;419(2):401–9. 19140805 10.1042/BJ20081482 PMC2741425

[16] Zhang L, et al. Gambogic acid inhibits Hsp90 and deregulates TNF-α/NF-κB in HeLa cells. Biochem Biophys Res Commun. 2010;403(3–4):282–7. 21074517 10.1016/j.bbrc.2010.11.018

[17] Zhao Z, et al. Steroid receptor coactivator-3 is a pivotal target of gambogic acid in B-cell Non-Hodgkin lymphoma and an inducer of histone H3 deacetylation. Eur J Pharmacol. 2016;789:46–59. 27370960 10.1016/j.ejphar.2016.06.048

[18] Kasibhatla S, et al. A role for transferrin receptor in triggering apoptosis when targeted with gambogic acid. Proc Natl Acad Sci U S A. 2005;102(34):12095–100. 16103367 10.1073/pnas.0406731102 PMC1189297

[19] Cheung EC, Vousden KH. The role of ROS in tumour development and progression. Nat Rev Cancer. 2022;22(5):280–97. 35102280 10.1038/s41568-021-00435-0

[20] Park MH, et al. Presenilin Mutation Suppresses Lung Tumorigenesis via Inhibition of Peroxiredoxin 6 Activity and Expression. Theranostics. 2017;7(15):3624–37. 29109765 10.7150/thno.21408 PMC5667337

[21] Thapa P, et al. The role of peroxiredoxins in cancer development. Biology (Basel). 2023;12(5):666. 37237480 10.3390/biology12050666 PMC10215932

[22] Krajarng A, et al. Apoptosis induction associated with the ER stress response through up-regulation of JNK in HeLa cells by gambogic acid. BMC Complement Altern Med. 2015;15:26. 25887496 10.1186/s12906-015-0544-4 PMC4340837

[23] Wu J, et al. Gambogenic acid induces apoptosis and autophagy through ROS-mediated endoplasmic reticulum stress via JNK pathway in prostate cancer cells. Phytother Res. 2023;37(1):310–28. 36086867 10.1002/ptr.7614

[24] Miller DR, Thorburn A. Autophagy and organelle homeostasis in cancer. Dev Cell. 2021;56(7):906–18. 33689692 10.1016/j.devcel.2021.02.010 PMC8026727

[25] Chen X, et al. Target identification of natural medicine with chemical proteomics approach: probe synthesis, target fishing and protein identification. Signal Transduct Target Ther. 2020;5(1):72. 32435053 10.1038/s41392-020-0186-y PMC7239890

[26] Nodwell MB, Sieber SA. ABPP methodology: introduction and overview. Top Curr Chem. 2012;324:1–41. 22160389 10.1007/128_2011_302

[27] Wang J, et al. A quantitative chemical proteomics approach to profile the specific cellular targets of andrographolide, a promising anticancer agent that suppresses tumor metastasis. Mol Cell Proteomics. 2014;13(3):876–86. 24445406 10.1074/mcp.M113.029793 PMC3945915

[28] Wang J, et al. Haem-activated promiscuous targeting of artemisinin in *Plasmodium falciparum*. Nat Commun. 2015;6:10111. 26694030 10.1038/ncomms10111 PMC4703832

[29] Wang J, et al. Mapping sites of aspirin-induced acetylations in live cells by quantitative acid-cleavable activity-based protein profiling (QA-ABPP). Sci Rep. 2015;5:7896. 25600173 10.1038/srep07896 PMC5379034

[30] Wang J, et al. In situ proteomic profiling of curcumin targets in HCT116 colon cancer cell line. Sci Rep. 2016;6:22146. 26915414 10.1038/srep22146 PMC4768257

[31] Luo P, et al. Mechanistic engineering of celastrol liposomes induces ferroptosis and apoptosis by directly targeting VDAC2 in hepatocellular carcinoma. Asian J Pharm Sci. 2023;18(6): 100874. 38149060 10.1016/j.ajps.2023.100874 PMC10749887

[32] Zhang Q, et al. Capsaicin ameliorates inflammation in a TRPV1-independent mechanism by inhibiting PKM2-LDHA-mediated Warburg effect in sepsis. Cell Chem Biol. 2022;29(8):1248-1259.e6. 35858615 10.1016/j.chembiol.2022.06.011

[33] Chen P, et al. Triptolide induces apoptosis and cytoprotective autophagy by ROS accumulation via directly targeting peroxiredoxin 2 in gastric cancer cells. Cancer Lett. 2024;587: 216622. 38246224 10.1016/j.canlet.2024.216622

[34] Luo P, et al. Celastrol induces ferroptosis in activated HSCs to ameliorate hepatic fibrosis via targeting peroxiredoxins and HO-1. Acta Pharm Sin B. 2022;12(5):2300–14. 35646542 10.1016/j.apsb.2021.12.007 PMC9136576

[35] Su Y, Chen L, Yang J. Hesperetin inhibits bladder cancer cell proliferation and promotes apoptosis and cycle arrest by PI3K/AKT/FoxO3a and ER stress-mitochondria pathways. Curr Med Chem. 2024. 10.2174/0109298673283888231217174702. 38357946 10.2174/0109298673283888231217174702

[36] Fang C, et al. IFN-γ-induced ER stress impairs autophagy and triggers apoptosis in lung cancer cells. Oncoimmunology. 2021;10(1):1962591. 34408924 10.1080/2162402X.2021.1962591 PMC8366549

[37] Zhu M, et al. Gambogic acid shows anti-proliferative effects on non-small cell lung cancer (NSCLC) cells by activating reactive oxygen species (ROS)-induced endoplasmic reticulum (ER) stress-mediated apoptosis. Med Sci Monit. 2019;25:3983–8. 31138775 10.12659/MSM.916835 PMC6559008

[38] Liu DD, et al. Celastrol exerts a neuroprotective effect by directly binding to HMGB1 protein in cerebral ischemia-reperfusion. J Neuroinflammation. 2021;18(1):174. 34372857 10.1186/s12974-021-02216-w PMC8353826

[39] Liu DD, et al. Target profiling of an anticancer drug curcumin by an in situ chemical proteomics approach. Methods Mol Biol. 2021;2213:147–61. 33270200 10.1007/978-1-0716-0954-5_13

[40] Gao L, et al. Targeting ROS-mediated crosstalk between autophagy and apoptosis in cancer. Adv Exp Med Biol. 2020;1260:1–12. 32304028 10.1007/978-3-030-42667-5_1

[41] B’Chir W, et al. The eIF2α/ATF4 pathway is essential for stress-induced autophagy gene expression. Nucleic Acids Res. 2013;41(16):7683–99. 23804767 10.1093/nar/gkt563 PMC3763548

[42] Bhardwaj M, et al. Regulation of autophagy by canonical and non-canonical ER stress responses. Semin Cancer Biol. 2020;66:116–28. 31838023 10.1016/j.semcancer.2019.11.007 PMC7325862

[43] Sun Y, et al. ent-Kaurane diterpenoids induce apoptosis and ferroptosis through targeting redox resetting to overcome cisplatin resistance. Redox Biol. 2021;43: 101977. 33905957 10.1016/j.redox.2021.101977 PMC8099784

[44] Snezhkina AV, et al. ROS generation and antioxidant defense systems in normal and malignant cells. Oxid Med Cell Longev. 2019;2019:6175804. 31467634 10.1155/2019/6175804 PMC6701375

[45] Sun T, et al. Gambogic acid impairs the maintenance and therapeutic resistance of glioma stem cells by targeting B-cell-specific *Moloney leukemia* virus insert site 1. Phytomedicine. 2024;135: 156070. 39326139 10.1016/j.phymed.2024.156070

[46] Zhang XW, et al. Thermal proteome profiling strategy identifies CNPY3 as a cellular target of gambogic acid for inducing prostate cancer pyroptosis. J Med Chem. 2024;67(12):10005–11. 38511243 10.1021/acs.jmedchem.4c00140

[47] Chi Y, et al. An open-labeled, randomized, multicenter phase IIa study of gambogic acid injection for advanced malignant tumors. Chin Med J (Engl). 2013;126(9):1642–6. 23652044

[48] Lei D, et al. An injectable gambogic acid loaded nanocomposite hydrogel enhances antitumor effect by reshaping immunosuppressive tumor microenvironment. Mater Today Bio. 2025;31: 101611. 40104652 10.1016/j.mtbio.2025.101611 PMC11919334

[49] Shan X, et al. Molecularly engineered carrier-free co-delivery nanoassembly for self-sensitized photothermal cancer therapy. J Nanobiotechnology. 2021;19(1):282. 34544447 10.1186/s12951-021-01037-6 PMC8454134

[50] Banik K, et al. Therapeutic potential of gambogic acid, a caged xanthone, to target cancer. Cancer Lett. 2018;416:75–86. 29246645 10.1016/j.canlet.2017.12.014

[51] Fahmy SA, et al. Emerging tendencies for the nano-delivery of gambogic acid: a promising approach in oncotherapy. RSC Adv. 2024;14(7):4666–91. 38318629 10.1039/d3ra08042k PMC10840092

[52] Liu Y, et al. Gambogic acid as a candidate for cancer therapy: a review. Int J Nanomed. 2020;15:10385–99. 10.2147/IJN.S277645 PMC7764553 33376327

